# Identification of Novel and Conserved miRNAs in Leaves of *In vitro* Grown *Citrus reticulata* “Lugan” Plantlets by Solexa Sequencing

**DOI:** 10.3389/fpls.2015.01212

**Published:** 2016-01-08

**Authors:** Rongfang Guo, Xiaodong Chen, Yuling Lin, Xuhan Xu, Min Kyaw Thu, Zhongxiong Lai

**Affiliations:** ^1^Department of Horticulture, Fujian Agriculture and Forestry UniversityFuzhou, China; ^2^Department of Horticulture, Institute of Horticultural Biotechnology, Fujian Agriculture and Forestry UniversityFuzhou, China; ^3^Institut de la Recherche Interdisciplinaire de ToulouseToulouse, France

**Keywords:** *in vitro* plantlet leaf, miRNA, *in vitro* conservation, miR398, *Citrus reticulate*

## Abstract

MicroRNAs (miRNAs) play essential roles in plant development, but the roles in the *in vitro* plant development are unknown. Leaves of ponkan plantlets derived from mature embryos at *in vitro* culture conditions were used to sequence small RNA fraction via Solexa sequencing, and the miRNAs expression was analyzed. The results showed that there were 3,065,625 unique sequences in ponkan, of which 0.79% were miRNAs. The RNA sequences with lengths of 18–25 nt derived from the library were analyzed, leading to the identification of 224 known miRNAs, of which the most abundant were miR157, miR156, and miR166. Three hundred and fifty-eight novel miRNA candidates were also identified, and the number of reads of ponkan novel miRNAs varied from 5 to 168,273. The expression of the most known miRNAs obtained was at low levels, which varied from 5 to 4,946,356. To better understand the role of miRNAs during the preservation of ponkan *in vitro* plantlet, the expression patterns of *cre-miR156a/159b/160a/166a/167a/168a/171/398b* were validated by quantitative real-time PCR (qPCR). The results showed that not only the development-associated miRNAs, e.g., *cre-miR156*/*159*/*166/396*, expressed highly at the early preservation period in the *in vitro* ponkan plantlet leaves but also the stress-related miRNAs, e.g., *cre-miR171* and *cre-miR398b*, expressed highly at the same time. The expression levels of most tested miRNAs were found to decrease after 6 months and the amounts of these miRNAs were kept at low levels at 18 months. After analyzing the expression level of their targets during the reservation of the ponkan *in vitro* plantlet, development-associated *cre-ARF6* and stress-related *cre-CSD* modules exhibited negative correlation with *miR167* and *miR398*, respectively, indicating an involvement of the miRNAs in the *in vitro* development of ponkan and function in the conservation of ponkan germplasm.

## Introduction

MicroRNAs (miRNAs) are the products of endogenous non-coding RNA genes, which perform regulatory roles in the development of plants and animals. MiRNAs are typically 18–25 nucleotide (nt) in length and exhibit different degrees of sequence complementary to target RNAs at the posttranscriptional level (Bartel, 2004; Jones-Rhoades et al., 2006). More evidences have shown that miRNAs play critical roles in plant cell division, morphogenesis, polarity development, hormone secretion, signal transduction as well as responses to stresses (Chen, 2005; Chuck et al., 2009; Liang et al., 2010; Wu et al., 2010; Zhao et al., 2010; Barciszewska-Pacak et al., 2015). Although deep sequencing to identify miRNAs has been reported in many plants, most of which belonged to model plants or main crops such as *Arabidopsis thaliana* (Hsieh et al., 2009), *Lycopersicon esculentum* (Moxon et al., 2008), *Oryza sativa* (Wei et al., 2011), *Zea mays* (Wang et al., 2011), *Triticum aestivum* (Meng et al., 2013), and *Sorghum bicolor* (Katiyar et al., 2015). There are more than 700 species in woody plants, among which fruits of citrus such as tangerine, citrange, tangelo, orange, pomelo, grapefruit, lemon, lime, ponkan, and citron are of great economic importance in the world. Ponkan (*Citrus reticulata*, an important citrus cultivar) is the major cultivar of mandarins in China. However, deep sequencing for discovery and identification of miRNAs was only reported in sweet orange flesh color and its rootstock, *Poncirus trifoliata* (synonym: *Citrus trifoliata*, but more commonly known as *Poncirus trifoliata*, which is not edible—a related genus plant of citrus rootstock; Song et al., 2010; Xu et al., 2010). Recently, deep sequencing of sRNA was also reported in embryogenic callus, leaf, flower, and fruit of sweet orange (Liu et al., 2014; Wu et al., 2015).

In the indoor preservation of citrus germplasm resources, the *in vitro* plantlets are used commonly for a longer subculture period and for relatively stable genetic characteristics compared with cell and tissue culture (Chen, 2011). In our Citrus Germplasm Resources Preservation Center, the *in vitro* plantlets are initiated from mature embryos and undergo a rapid growth phase and then enter a long and slow-growing period in the preservation. Leaf *in vitro* morphogenesis is an indicator of plant health in our preservation system. Investigations on *in vivo* leaves indicate that miRNAs are involved in the plant development (Juarez et al., 2004; Ori et al., 2007; Meng et al., 2013). There is no clue about how miRNAs regulate the development of leaf in *in vitro* ponkan plantlets. In the present study, the *in vitro* leaves of ponkan were used as materials for discovery and identification of novel and conserved miRNAs by Solexa deep sequencing.

Solexa sequencing provides solid information of the sequences and whether the length of sequences is in accordance with the length of miRNA (Xie et al., 2011). In order to clarify how miRNAs and their targets influence the process of leaf development *in vitro*, we established a small RNAs library with mixed leaf samples from the early preservation period covering three stages of leaf development, i.e., 9 days after germination (DAG) corresponding to the initiation stage, 22 DAG of fast-growing stage, and 27 DAG of stable-growing stage, and identified miRNAs and their targets in ponkan *in vitro* plantlet. To further investigate the role of miRNA in the process of *in vitro* ponkan preservation, the expression patterns of miRNA and their targets of the leaves sampled, both in 6 months after germination (MAG) corresponding to the early preservation period and in 18 MAG corresponding to the slow-growing stage in the later preservation period, were also detected by means of quantitative real-time PCR (qPCR).

## Materials and methods

### Plantlets and growth conditions

Fruits of ponkan (*C. reticulata* “Lugan”) were picked from orchard in Youxi in Youxi Technology Bureau (Fujian Agriculture and Forestry University has approved the study. The land accessed is not privately owned and protected. No protected species were sampled. No specific permissions were required for these locations/activities. The Youxi Technology Bureau is located in Jiefang Road No. 17 in Youxi County, Sanming City). The seeds were immersed in soap water for 3–5 min to get rid of the mucilage on the seeds, then drained and washed with distilled water until they reached neutral pH. Subsequently, the ponkan seeds were sterilized for 30 s in 75% ethanol and washed with sterile water until they reached neutral pH, and then immersed in 0.1% mercuric chloride solution for 8 min, followed by washing five times with sterile water. The episperm and endopleura of seeds were cut and the embryos were placed in culture flask (6 × 9 cm) with solid growth medium [sterilized Murashige-Skoog (MS) salt solution (pH = 5.8) + 0.6% agar + 3% sucrose]. Plants were grown under a photoperiod of 12 h light/12 h dark (1200 lx) in a plant growth chamber at 25°C (Chen, 2011). Finally, 9 DAG, 22 DAG, 27 DAG, 6 MAG, and 18 MAG old leaves of *in vitro* ponkan plantlets were collected for measurements. Leaf samples were rapidly and gently collected from the surface of the filter paper. Small RNA was isolated from 9 DAG, 22 DAG, and 27 DAG old leaves and used for Solexa sequencing.

### Construction of ponkan *In vitro* plantlet leaf small RNA library and solexa sequencing

Total RNA was isolated from the samples mentioned above using TRIzol Reagent kit (Invitrogen, Life Technologies, Carlsbad, CA) according to the manufacturer's instructions and pooled in an equal fraction ratio for the construction of ponkan small RNA library. After that, the small RNA fragments of 16–30 nt were isolated from the 15% PAGE gel and purified, and then the small RNAs were ligated to a 5′ RNA adapter (5′-GUUCAGAGUUCUACAGUCCGACGAUC-3′) and a 3′ RNA adapter (5′-pUCGUAUG CCGUCUUCUGCUUGidT-3′; p, phosphate; idT, inverted deoxythymidine) sequentially using T4 RNA ligase. The samples were reversely transcribed to cDNA with the RT primer (5′-CAAGCAGAAGACGGCATACGA-3′) using Superscript II reverse transcriptase (Invitrogen), and amplified by PCR. Finally, the small RNAs from samples were sequenced by using Solexa sequencing technology (Beijing Genomics Institute, China) and submitted to SRA with the accession number SRP066915.

### Analysis of small RNA of ponkan *In vitro* plantlet leaf

In order to predict the potentially conserved and novel miRNAs of ponkan *in vitro* plantlet leaf, bioinformatics analysis of the Solexa sequencing data was conducted. Firstly, the 35-nt sequence tags from Solexa sequencing gone through the data were cleaned, which eliminated several kinds of contaminants and the low-quality tags. The small RNA tags were mapped to the *Citrus clementina* genome (http://www.phytozome.net/clementine.php) using Short Oligonucleotide Analysis Package (SOAP) to analyze their expression and distribution in the genome (Li et al., 2008). Secondly, the cleaned tags were compared against non-coding RNAs from Rfam database (http://www.sanger.ac.uk/software/Rfam) and the NCBI GenBank database (http://www.ncbi.nlm.nih.gov/) to classify the degradation fragments of non-coding RNAs. Any small RNAs having exact matches with the degradation fragments were excluded from further analysis. In addition, the unique sRNA sequences were used for searching miRNA sequences using miRBase 21 (http://mirbase.org/) to identify the known miRNAs in ponkan *in vitro* plantlet leaf. Finally, the novel miRNA of the ponkan *in vitro* plantlet leaf was predicted from the surplus unannotated small RNAs using MIREAP (http://sourceforge.net/projects/mireap/), and the parameters setting for the identification of novel miRNA are minimal miRNA length: 18, maximal miRNA length: 25, minimal miRNA (reference) length: 20, maximal miRNA (reference) length: 23, uniqueness of miRNA: 20, maximal energy: −18, minimal space: 5, maximal space: 300, minimal mature pair: 16, maximal mature bulge: 4, maximal duplex asymmetry: 4, and flank sequence length: 20.

### Prediction of targets of ponkan *In vitro* plantlet leaf miRNAs

It is necessary to identify and characterize the targets of miRNAs in order to understand the biological functions of them. In the present experiment, the potential targets of ponkan miRNAs were predicted using the psRobot (http://omicslab.genetics.ac.cn/psRobot/) and TargetFinder (http://targetfinder.org/)program (Kiełbasa et al., 2010; Wu et al., 2012). Newly identified ponkan miRNAs sequences were used as custom miRNA sequences and *C. clementina* genome (http://www.phytozome.net/clementine.php) sequences were used. The rules used for predicting ponkan novel miRNAs targets are (1) no more than four mismatches between the sRNA and the target (G-U bases count as 0.5 mismatches); (2) no more than two adjacent mismatches in the miRNA–target duplex; (3) no adjacent mismatches in positions 2–12 of the miRNA–target duplex (5′ of miRNA); (4) no mismatches in positions 10–11 of miRNA–target duplex; (5) no more than 2.5 mismatches in positions 1–12 of the of the miRNA–target duplex (5′ of miRNA); (6) minimum free energy (MFE) of the miRNA–target duplex should be ≥75% of the MFE of the miRNA bound to its perfect complement.

### Real-time quantitative PCR of ponkan miRNAs and their targets

The qPCR was used to validate results obtained from high throughput sequencing of ponkan miRNAs and their targets. RNA samples from the five embryogenic cultures described above were reverse transcribed using an One-Step PrimeScript miRNA cDNA Synthesis Kit (Perfect Real Time) (Takara Code: D350A) and PrimeScript® RT reagent Kit (Takara Code: DRR037A) for miRNA and target genes tested, respectively. Expression profiles of 13 miRNAs and six targets were examined using an SYBR® PrimeScript™ miRNA RT-PCR Kit (Takara Code: RR716) and SYBR® Premix Ex Taq™ II (Tli RNaseH Plus) (Takara Code: RR820A), respectively. All reactions were performed in triplicate in a LightCycler 480 qPCR instrument (Roche Applied Science, Switzerland) with a dissociation curve used to control for primer dimers in the reactions. Mature miRNA abundance was calculated relative to expression of reference genes cre-U6 snRNA; miRNA and target names and primer sequences are provided in Supplementary Table S4.

### Statistical analysis

Statistical analysis was performed using the SPSS package program version 11.5 (SPSS, Chicago, IL, USA). The data were analyzed by one-way analysis of variance. The values are reported as means ± standard error (SE) for all results. Differences were considered significant at *P* < 0.05.

## Results

### Categories and size distribution of small RNAs in ponkan

**Figure 1:**
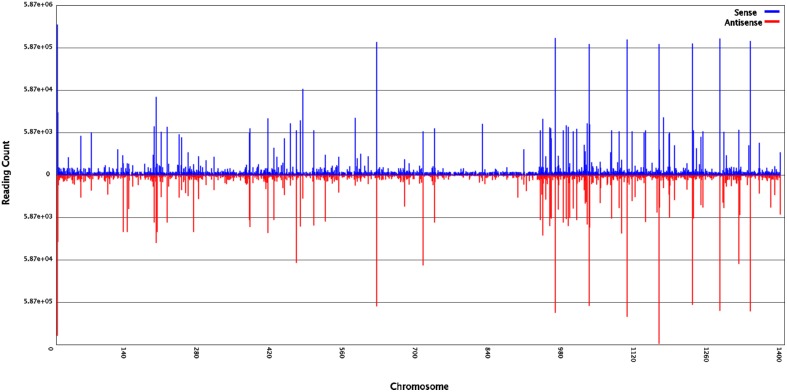
**Small RNAs of ponkan ***in vitro*** plantlet leaf distribution across different ponkan chromosomes**.

To identify miRNAs involved in the development of ponkan *in vitro* plantlet leaf, a sRNA library was generated from the leaves of ponkan *in vitro* plantlet (three stages of pooled samples) and sequenced by a Solexa/Illumina analyzer. In total, 19,145,956 raw reads were generated, including 180,152 low-quality reads and 18,965,804 high-quality reads. After removing low-quality reads and adapter contaminants, 18,033,510 clean reads with lengths ranging from 18 to 30 nt were obtained from the leaves of ponkan *in vitro* plantlet (Table 1). According to the general principle of miRNA analysis, sRNAs were first compared with *C. clementina* (http://www.phytozome.net/clementine.php) and then the number of sRNA sequences matching with the genome was identified (Figure 1). After SOAP analysis, 15,865,321 (87.98%) sequences were found to match with the genome perfectly, which contained 2,018,630 (65.85%) unique sequences.

**Table 1:** **Distribution of small RNAs among different categories in ponkan ***in vitro*** plantlet**.

**Category**	**Unique sRNA**	**Percent (%)**	**Total sRNA**	**Percent (%)**
Total	3065625	100.00	18033510	100.00
Exon-antisense	55429	1.81	175677	0.97
Exon-sense	91444	2.98	301569	1.67
Intron-antisense	38444	1.25	93759	0.52
Intron-sense	59701	1.95	241971	1.34
miRNA	24179	0.79	9436789	52.33
rRNA	64266	2.10	881150	4.89
Repeat	316430	10.32	721841	4.00
snRNA	1748	0.06	4499	0.02
snoRNA	1063	0.03	1894	0.01
tRNA	6487	0.21	262679	1.46
Unann	2406434	78.50	5911682	32.78

Among the unique sequences, 24,179 (0.79%) were found to be similar with the known miRNAs after comparing against all plant miRNA precursors and mature miRNAs in miRBase 21. Other unique sequences, including ribosomal RNA (rRNA, 64,266, 2.10%), small nuclear RNA (snRNA, 1748, 0.06%), small nucleolar RNA (snoRNA, 1063, 0.03%), and transfer RNA (tRNA, 6487, 0.21%), were also identified in ponkan *in vitro* plantlet leaves by performing a BLASTN search against the Rfam (12.0) database (http://rfam.xfam.org/) (Nawrocki et al., 2014). However, 2,406,434 (78.50%) unique sequences could not be annotated (Table 1).

**Figure 2:**
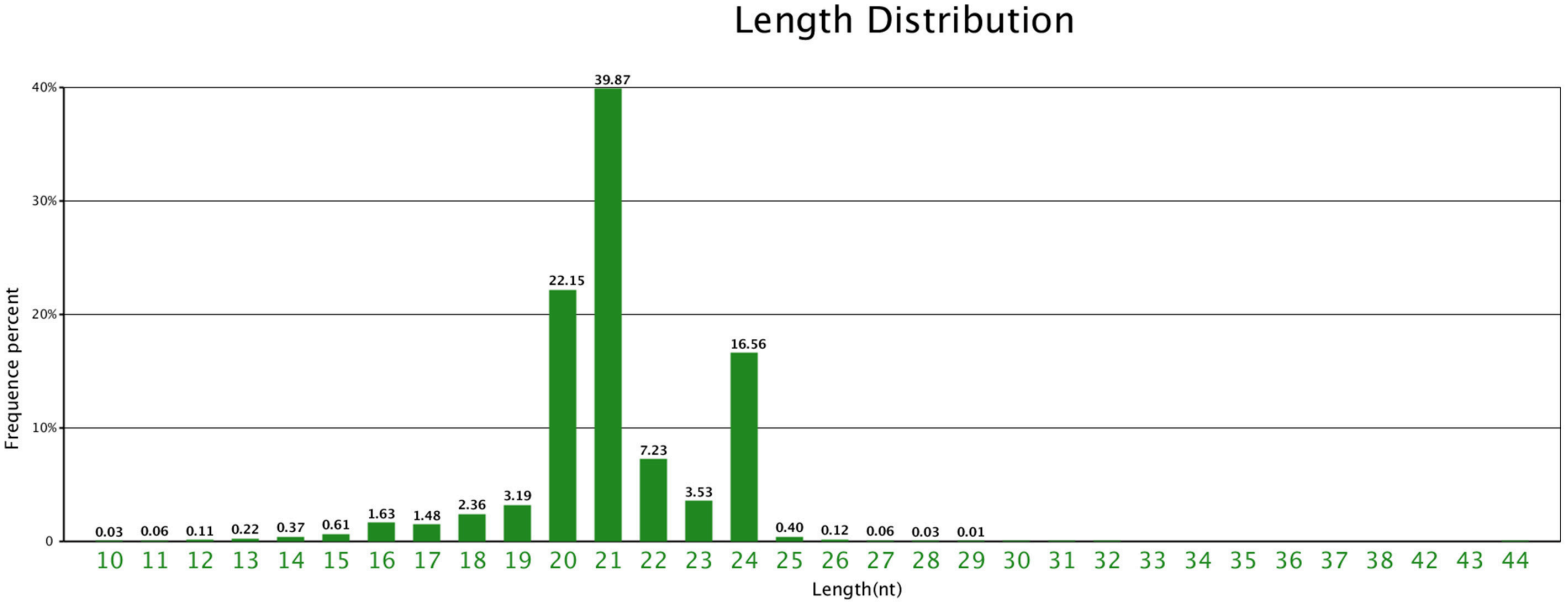
**Length distribution of unique sequences in ponkan ***in vitro*** plantlet**.

The size distribution patterns of the sRNAs' unique sequences in ponkan were listed in Figure 2. The results showed that the size distribution of sRNAs was uneven. The length of most unique sRNAs varied from 18 to 25 nt, and 39.87% were 21 nt in size, followed by 20 nt (22.15%) and 24 nt (16.56%).

### Identification of known miRNAs in ponkan

In order to identify the conserved miRNAs from ponkan sRNA sequences, the unique sRNAs were compared with miRNA precursors or mature miRNAs of all plant data in miRBase 21. A total of 24,179 sequences were found to be orthologs of known miRNAs in other plant species. After Blastn, 224 known miRNAs, belonging to 201 miRNA families, were identified from the leaf of ponkan *in vitro* plantlet (Supplementary Table S1). There were 178 miRNA families containing only one member, whereas 23 miRNA families contained two members.

The length distribution of known miRNAs in ponkan was listed in Supplementary Table S1, which varied from 18 to 24 nt. The lengths of most miRNAs were 21 nt (40.41%), followed by 24 nt (20.54%) and 22 nt (10.27%).

The expression frequency of known miRNAs in ponkan was found to have large divergences ranging from 5 to 4,946,356. Most of the known ponkan miRNAs (66.52%) were sequenced < 1000 times, and only 2.01% known miRNAs (miR530, miR3623, and miR7757) were sequenced < 10 times. Generally, the 224 known miRNAs were classified into two categories based on their conservation, and conserved miRNAs expressed higher than non-conserved miRNAs. MiR157 was the most abundant one with 4,945,356 reads detected in leaves, followed by miR156 and miR166. High accumulation of miR157 indicated its important roles in the development of ponkan *in vitro* plantlet leaves. Non-conserved miRNAs such as miR3954 and miR5770 were also highly expressed (177,692 and 110,104, respectively).

### Prediction of novel miRNAs in ponkan

The unannotated sequences were mapped with the genome sequences of *C. clementina* (http://www.phytozome.net/clementine.php), and the perfectly matched sequences were collected to predict the secondary structure of each locus using the software MIREAP. Analyzed sRNAs are considered as candidate miRNA genes only if they fulfill the following criteria: (1) a mature sequence localized in one arm of the stem-loop structure and between 19 and 24 nt; (2) the corresponding miRNA^*^ sequence identified; (3) the pre-miRNA sequence folded into an appropriate stem-loop hairpin secondary structure; (4) the mfe of secondary structures ≤ −20 kcal/mol; and (5) no more than 7-nt mismatches in the miRNA:miRNA^*^ duplex (Meyers et al., 2008). The results revealed that 358 sRNA sequences that perfectly matched citrus genome were able to fold into stem-loop hairpin secondary structures. The number of reads of ponkan novel miRNAs varied from 5 to 168,273, among which *cre-miR-m0019* was expressed at the highest level (168,273), followed by *cre-miR-m0190* and *cre-miR-m0175*. Only 5.31% novel miRNAs were expressed over 1000 times (Table 2, Supplementary Table S2).

**Table 2:** **Ten novel miRNAs identified from ponkan ***in vitro*** plantlet**.

**Novel miRNA**	**Location**	**Mfe**	**Count**	**Sequence**	**Length (nt)**	**5′/3′**
Cre-miR-m0019	scaffold_1:15209053:15209175: −	−55	168238	GTGACAGAAGATAGAGAGCGC	21	5′
Cre-miR-m0190	scaffold_5:5960222:5960542: −	−75	39182	GCAATGCTCTTGAAGGACTAC	21	3′
Cre-miR-m0175	scaffold_5:5960245:5960485: +	−59	35081	AAGTCATTAGAAGAACTGCCG	21	5′
Cre-miR-m0053	scaffold_2:8093618:8093764: −	−78	7107	CCGCAGGGGCGACATGAGATC	21	5′
Cre-miR-m0144	scaffold_4:3919238:3919484: +	−94	5367	ACTGACAGCGGCTGTACTGTAGT	23	5′
Cre-miR-m0094	scaffold_3:25587984:25588086: +	−38	3689	TGCTTGTTGATTGTCATCTAA	21	5′
Cre-miR-m0133	scaffold_3:46661840:46661982: −	−53	2946	CATGTTTCGGGTTTGTGCGTG	21	5′
Cre-miR-m0106	scaffold_3:50445048:50445335: +	−85	2416	GGTGTCGTGGTGTAGTTGGTT	21	5′
Cre-miR-m0073	scaffold_2:28637496:28637638: −	−53	1574	GGTCATGGGAGGATTGGCGA	20	5′
Cre-miR-m0238	scaffold_6:20198376:20198482: +	−66	2038	CTGGATGCAACTGTGGTACGG	21	3′

According to Mfold, the precursors of the miRNAs had negative folding free energies ranging from −18.10 to −131.00 to 116.60 kcal/mol. The average Mfold was −45.16 kcal/mol (Table 2).

**Figure 3:**
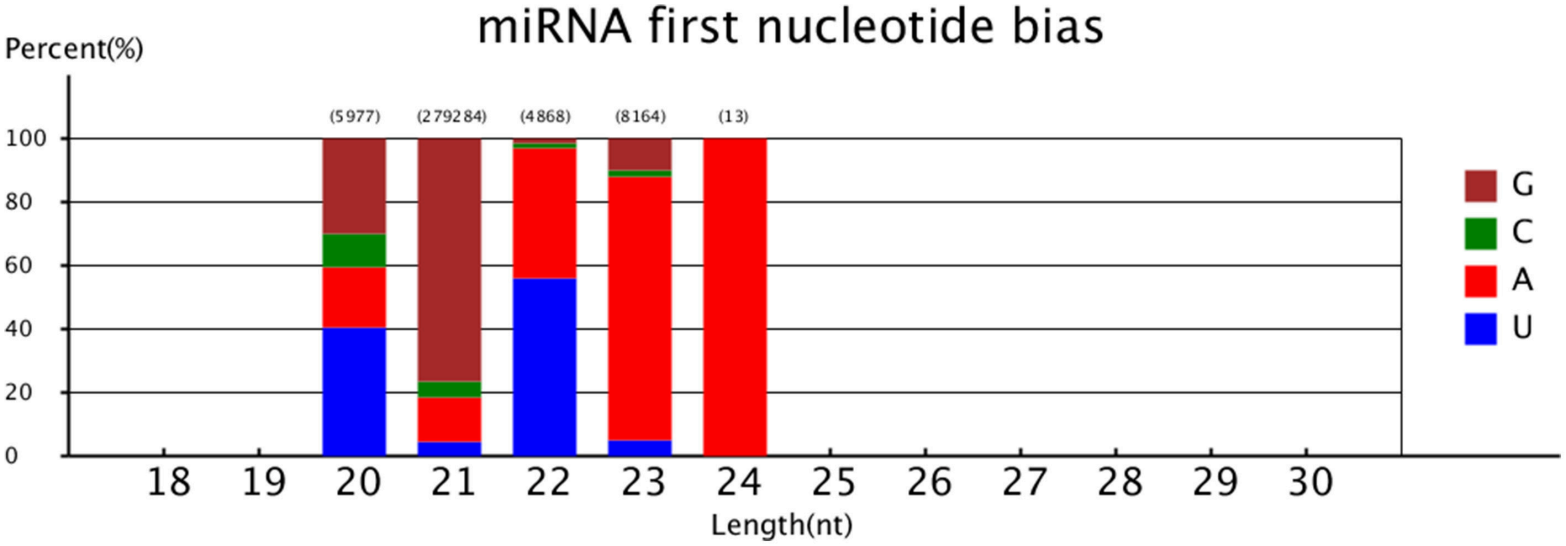
**MiRNA first nucleotide bias**.

The first nucleotide bias of the different lengths' novel miRNA was also analyzed. The results showed that > 40% miRNAs of 20 and 22 nt began with 5′ uridine, and only <5% miRNAs of 20 and 23 nt began with 5′ uridine (Figure 3).

### Prediction of target genes of miRNA in ponkan

**Figure 4:**
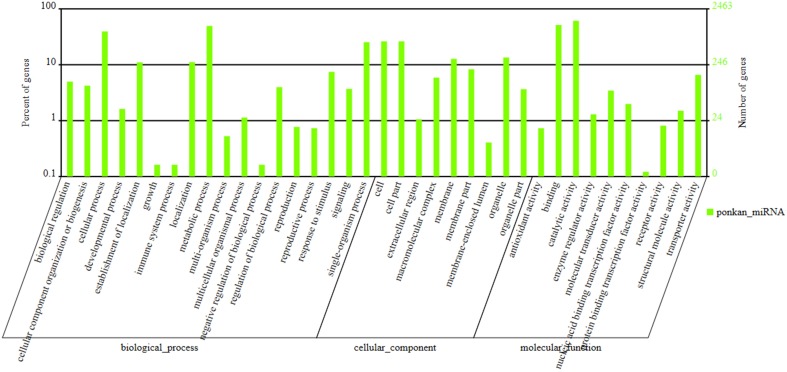
**GO (Gene ontology) annotation of ponkan miRNA**.

The potential targets of known miRNAs of ponkan were predicted using the psRobot and TargetFinder software. A total of 202 genes and their 7594 corresponding target genes were predicted. Among these genes, 184 known miRNAs and their 2895 corresponding target genes were acquired using psRobot, and 197 known miRNAs and their 5134 corresponding target genes were found after processed with TargetFinder. There are 1398 target genes in common between psRobot and TargetFinder. For novel miRNAs, targets of 358 novel miRNAs were predicted, and targets of 265 novel miRNAs including 4738 corresponding target genes were consistent with the features of target according to psRobot and TargetFinder predictions (Figure 4).

GO (Gene Ontology) annotation collected from Gene ontology (http://geneontology.org/) and NCBI (http://www.ncbi.nlm.nih.gov/ (Gene ontology)) database information were also conducted in our study. Gene annotation and classification in accordance with the biological pathway (Biology Process), cellular localization (Cellular component), and molecular function (Molecular Function) were listed in Figure 4. Two thousand four hundred and sixty-three genes were classified into 18 biology processes, nine cellular components, and 10 molecular functions. Among these, 1508 genes lied in catalytic activity, 1268 genes in binding section, and 1220 genes in metabolic process.

After enriched analysis in biology process, lignin metabolic process (GO: 0009808) ranked first with 15 differently expressed genes out of 19 genes in this term after comparing its cluster frequency with the genome frequency followed by dicarboxylic acid metabolic process (GO: 0043648) and inorganic anion transport (GO: 0015698). Glutamate receptor activity (GO: 0008066), malate dehydrogenase activity (GO: 0016615), and ion channel activity (GO: 0005216) were the three molecular functions that enriched strongest. For cellular location, AP-type membrane coat (GO: 0030119) and chloroplast outer membrane (GO: 0009707) were the strongest enriched location (Supplementary Table S3).

### Development of ponkan *In vitro* plantlet in the process of conservation

**Figure 5:**
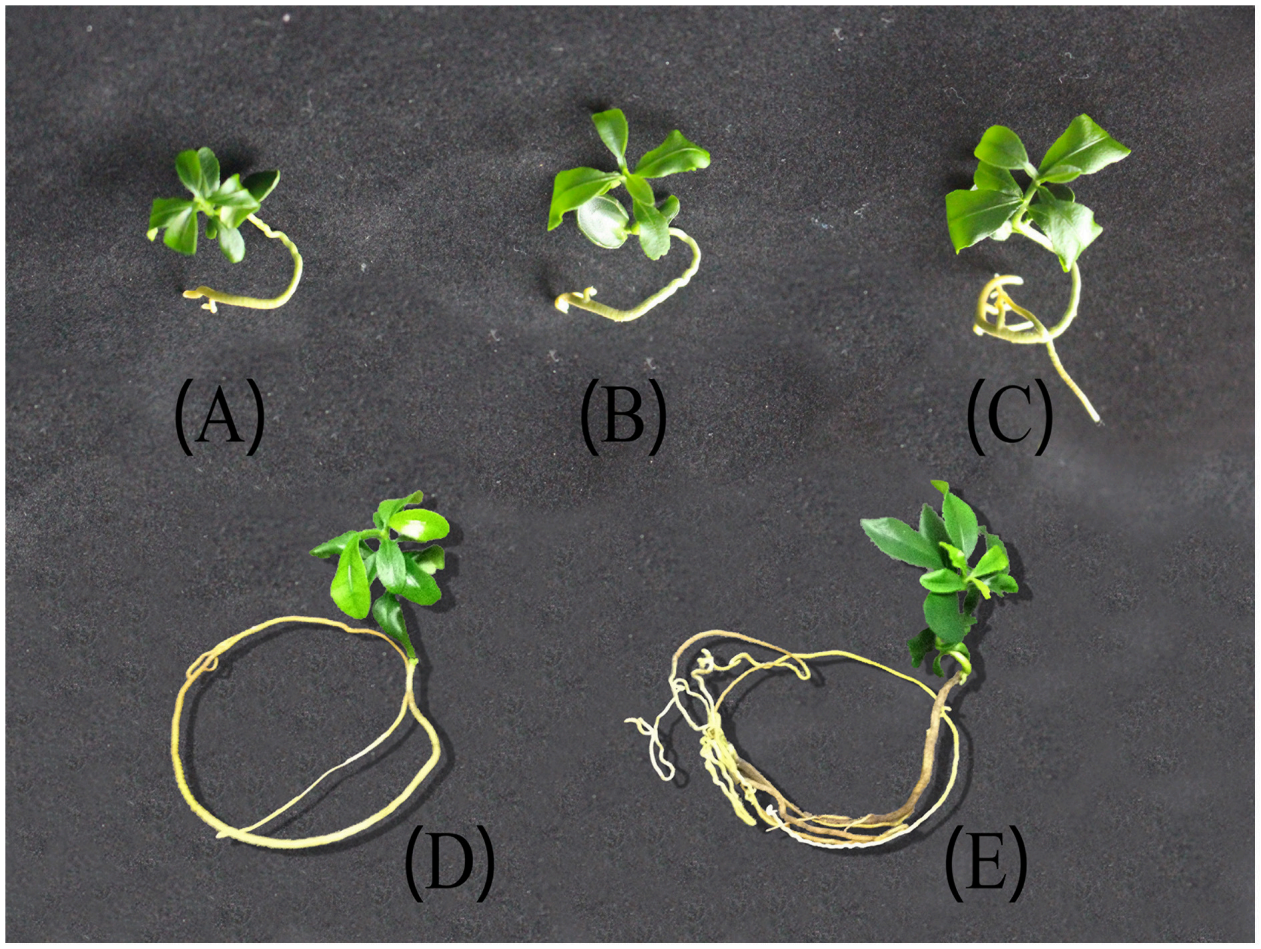
**Ponkan plantlets under different storage time**. **(A)** 9 days after germination (DAG) corresponding to the initiation stage, **(B)** 22 DAG of fast-growing stage, **(C)** 27 DAG of stable-growing stage, **(D)** 6 months after germination (MAG), **(E)** 18 MAG. Both **(D,E)** were corresponding to slow-growing stage.

After the mature embryos were incubated on MS and cultured for 4 days, the radicle emerged, and the shoot started to grow after 3 days. After another 9 days, a 4–5 leaf plantlet (9-day-old leaf) was formed. When the leaf was 22 days old, the area was about twice that of a 9-day-old leaf. The area of 27-day-old leaf was as much as that of a 22-day-old leaf and the color became dark green. After 6 months, the growth of plantlet was very slow and the leaf color became darker and darker. In 18 months, the leaf thickness increased and the leaf area enlarged (Figure 5). During the preservation, the ponkan plantlets suffered from nutrient and water stresses. Leaf was the most sensitive organ that reflected the growth status of the *in vitro* plantlets. Thus, leaves were sampled in the present investigation.

### Expression patterns of miRNAs and targets in the preservation of ponkan

**Figure 6:**
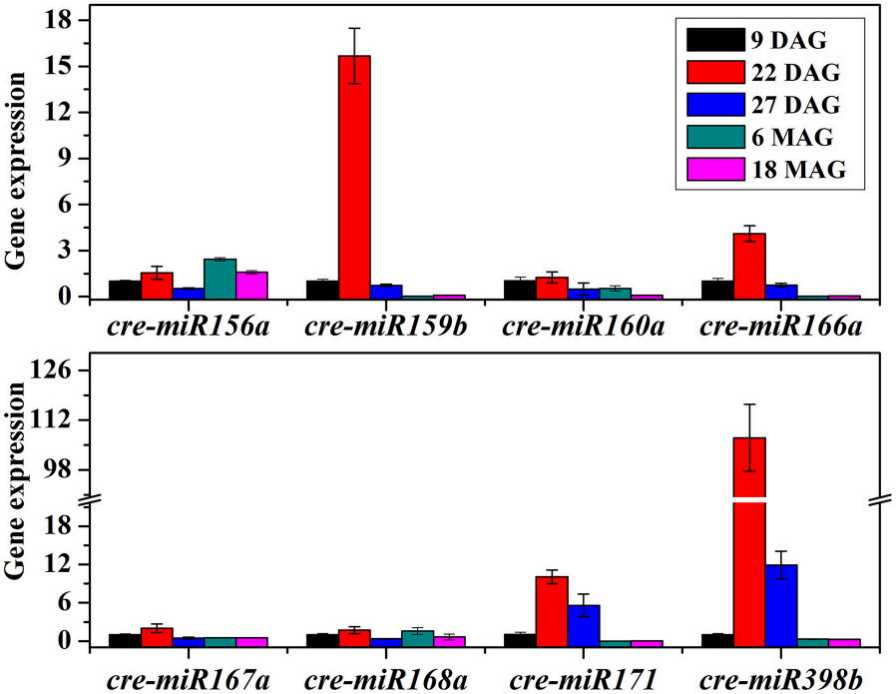
**QPCR analysis of relative expressions of miRNAs in ponkan ***in vitro*** plantlet leaf**.

**Figure 7:**
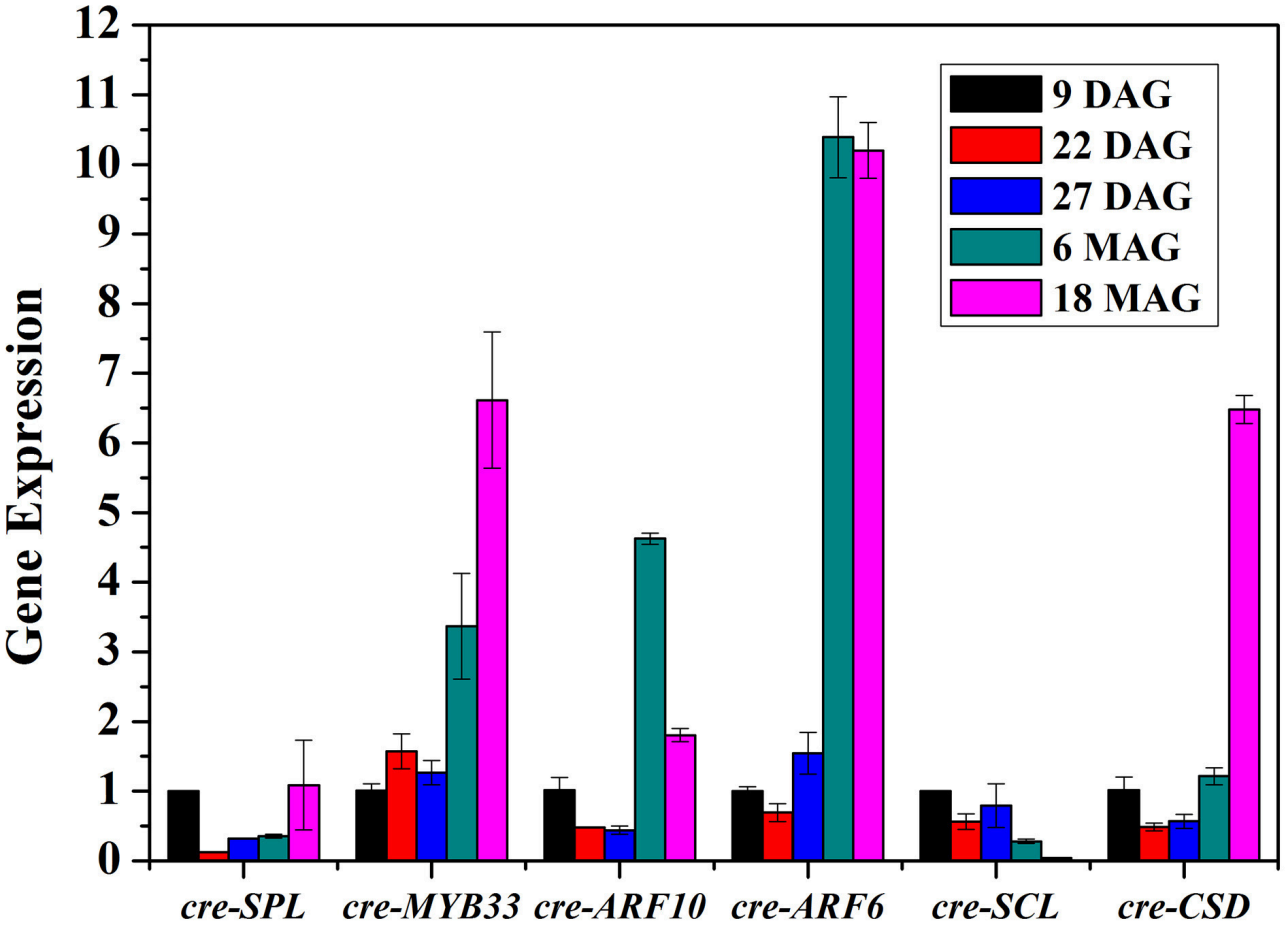
**QPCR analysis of relative expressions of the targets of miRNAs in ponkan ***in vitro*** plantlet leaf**.

To better understand the role of miRNAs during the preservation of ponkan *in vitro* plantlet, the expressions of miRNA and some target genes in 9 DAG, 22 DAG, 27 DAG, 6 MAG, and 18 MAG in ponkan *in vitro* plantlet leaf were analyzed using qPCR (Figures 6, 7).

The results showed that the tested miRNAs were expressed at various levels in the early preservation period, including the initial phase and fast-growing stage of the *in vitro* ponkan plantlets, i.e., 9 & 22 DAG, and the stable-growing stage, i.e., 27 DAG. The expression pattern can be divided into three categories (Figure 6): (i) the expression levels of *cre-miR159b, cre-miR166a, cre-miR171*, and *cre-miR398b* first reached the highest level in 22 DAG. Then, during the stable-growing stage, i.e., 27 DAG, the expression levels of *cre-miR171, cre-miR398b, cre-miR159b*, and *cre-miR166a* decreased. (ii) The expression levels of *cre-miR156a, cre-miR167a*, and *cre-miR168a* did not change significantly from 9 to 22 DAG, and then decreased in 27 DAG. (iii) The expression levels of *cre-miR160a* and *cre-miR167a* did not change significantly during 9–27 DAG. After six MAG, the *in vitro* plantlets arrived at the slow-growing stage, which was corresponding to the later preservation period, in which most of the tested miRNAs expressed at lower levels. The decreased expression level of *cre-miR156a* seemed postponed.

The expressions of *cre-SPL, cre-MYB33, cre-ARF10, cre-ARF6, cre-SCL*, and *cre-CSD*, target genes of *cre-miR156, cre-miR159, cre-miR160, cre-miR167*, and *cre-miR171*, respectively, were at varied levels (Figure 7). The amounts of *cre-SPL* and *cre-SCL* were kept at low level during the conservation of ponkan *in vitro* plantlet. Other target genes such as *cre-MYB33, cre-ARF10, cre-ARF6*, and *cre-CSD* exhibited similar expression pattern, decreased at the early stage, and increased at the later stage except unchanging *cre-MYB33* level at the early stage.

## Discussion

### A complex miRNA population existed in ponkan *In vitro* plantlet leaf

It is well known that miRNA play key roles in the development of leaf. In the present study, a total of 3,065,625 unique sequences containing 224 conserved miRNAs and 358 novel miRNAs were detected. It was obvious that the percentage of sRNAs with 21 nt in length was much higher than that with 24 nt in ponkan. Similar results were found in wheat (Yao et al., 2007), populus (Barakat et al., 2007), and tomato (Moxon et al., 2008), and the length distribution of sRNAs was mainly at 21 nt. The sRNAs from leaf, flower, and fruit in sweet orange showed that 24 nt sRNAs were the most abundant (Liu et al., 2014), which was also found in the majority of angiosperms (Morin et al., 2008; Szittya et al., 2008; Chen et al., 2009; Donaire et al., 2011; Lin and Lai, 2013). The size distribution of known miRNAs was mainly at 21 nt, thereby accounting for 42.41%, and 20-nt miRNA take the proportion of 8.48%. Although 20-nt miRNA does not represent a major class of ponkan miRNAs, highly conserved 20-nt miRNAs appear in most plant species. miR156 and miR394 are predominantly 20-nt long in many land plants, which were also found in ponkan *in vitro* plantlet leaves.

There were many conserved miRNAs in angiosperm plants. In the present study, most of the identified miRNA families in ponkan also existed in other plant species. For example, cre-miR156 family had a perfect match with miR156 in other plants, such as *Arabidopsis thaliana, Oryza, Sorghum, Medicago, Populus, Gossypium*, and *Vitis*. The cre-miR160/166/396 families were also found to be highly homologous to those forms in other plants. Furthermore, there were some specific miRNAs belonging to monocotyledon or dicotyledon. For example, miR444 was regarded as monocot-specific miRNAs (Yao et al., 2007; Sunkar and Jagadeeswaran, 2008; Zhang et al., 2009), whereas we detected the expression of miR444 in ponkan. On the other hand, miR158/163/173/403/472/479 was considered to be dicot-specific miRNAs (Jones-Rhoades and Bartel, 2004; Yin and Shen, 2010), which were also found in our experiment except for miR163. The inexistence of miR163 may be because of severely repressed leaves (Ha et al., 2009).

### The regulatory role of miRNAs during the development of ponkan *In vitro* plantlet leaf

Leaf was the first lateral organ produced by the activity of shoot apical meristem. Leaf development is a multifaceted process during which a small group of undifferentiated cells get recruited in the meristem (Tsukaya, 2006). A complicated regulatory network involved in transcription factors and hormones is necessary to ensure the process of leaf development on the rails. To ensure the normal operation of various physiological processes of organisms, a tight regulatory network must operate precisely, which is constituted by the interaction of miRNAs and their target genes (Alvarez-Garcia and Miska, 2005). Some miRNAs have been found to play critical roles during the development of leaf in recent years, such as miR156, miR159, miR160, miR166, and miR396. In the conditions of present *in vitro* preservation, cre-miR156, cre-miR159, cre-miR160, cre-miR166, and cre-miR396 were all detected. The most abundant miRNAs belong to the families miR157, followed by miR156, miR166, and mir167, which are described as the most widespread miRNA families in plants (Sun, 2012). However, in leaf of cork oak (*Quercus suber*), the most abundant is miR167 and not miR157, and miR167 is one of the miRNA families involved in the regulation of auxin signaling.

MiR157 was predicted to target squamosa promoter-binding protein (SBP) or squamosal promoter binding-like protein (SPL) to function in multiple stress responses in tomato (Luan et al., 2014). In our study, the abundant reads of miR157 were also detected in ponkan *in vitro* plantlet leaves, indicating its key role in preservation of citrus germplasm resources.

**Figure 8:**
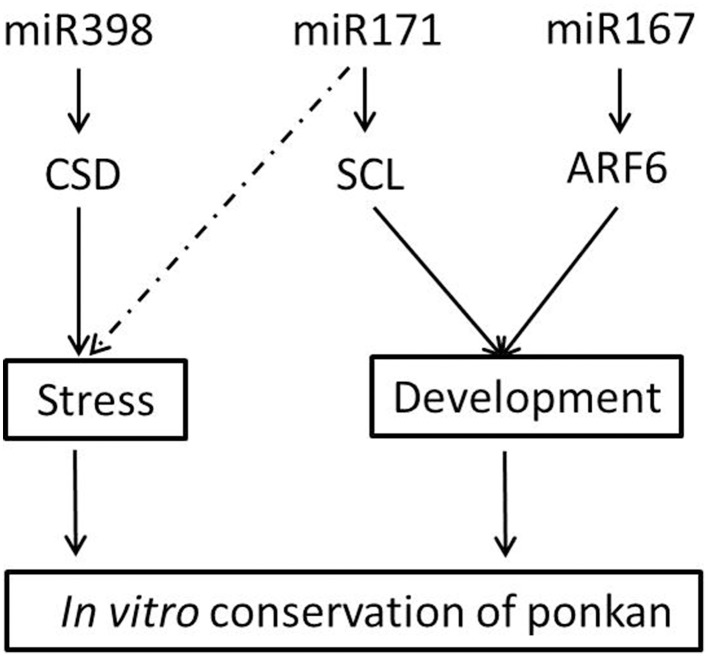
**Possible roles of miRNA in the ***in vitro*** conservation of ponkan**.

MiR167 targets the members of AUXIN RESPONSE FACTOR (ARF) family of transcription factors, namely ARF6/8, involved in early auxin response through activation of auxin-responsive genes (Wu et al., 2006), as well as IAA-Ala Resistant3 (IAR3), which hydrolyzes an inactive form of auxin (Kinoshita et al., 2012). The regulation of auxin signaling pathways by miR167 and its targets has been related to male and female flower development (Wu et al., 2006) and osmotic stress-induced root architecture changes (Kinoshita et al., 2012). In our study, the expression of *cre-ARF6* was down-regulated at nine DAG, and afterwards, it was up-regulated until 18 MAG, which is consistent with the first high and then low expression level of *cre-miR167*, indicating that miR167–ARF6 was involved in the regulation of leaf development of ponkan *in vitro* plantlet leaf (Figure 8).

Beside miR167 in ponkan, miR156 is one of the most abundant and evolutionarily conserved miRNAs in plants, which regulated plastochron length by quantitatively modulating the levels of *SQUAMOSA PROMOTER BINDING PROTEIN-LIKE* (*SPL*) transcription factors (Schwarz et al., 2008; Wang et al., 2008). Overexpression of miR156 accelerated the rate of leaf initiation. A similar effect was observed in the *spl9 spl15* double mutant, indicating that *SPL* genes are targeted by miR156 in *Arabidopsis*. SPL proteins could modulate auxin accumulation or response (Bencivenga et al., 2012). In our study, the expression of *cre-SPL* was down-regulated from 22 DAG to 6 MAG. This could be due to the different function of SPL. It was reported that the miR156-SPL module could be responsible for the lateral root development (Yu et al., 2015). The root development in ponkan *in vitro* plantlet is different from that grown in outdoor environment. The same conflicting expression trends were also observed in the expression of *cre-miR159-MYB33* and cre-*miR160-ARF10*.

### Stress-responding miRNAs were involved in the preservation of ponkan *In vitro* plantlet

MiRNAs not only function as plant developmental regulators but also play important roles in adaption to stress conditions. MiR171 can regulate shoot branching through targeting GRAS gene family members *SCARECROW-LIKE6* (*SCL6*) in *Arabidopsis* (Wang et al., 2010). Transgenic plants overexpressing *Arabidopsis miR171a* have been reported to exhibit multiple developmental defects, including reduced cauline leaf and rosette leaf formations and reduced shoot branching (Wang et al., 2010). In our study, the high expression level of *cre-miR171* combined with the low expression level of *cre-SCL* during the early stage indicated that miR171-SCL might mainly act as a leaf formation regulator at the early stage as the leaf area is expanding and the number of blades is increasing (Figure 8). After reserved for 6 months up to 18 months, the growth of *in vitro* ponkan plantlet decreased, and *cre-SCL* exhibited a very low expression level. At the same time, the expression level of *cre-miR171* was very low possibly due to stress from the limited nutrition and environment. It was reported that the expression of *miR171* was involved in drought response in *Solanum tuberosum* and can be regulated by light, temperature, and salt stress (Hwang et al., 2011).

MiR398 is required for embryo morphogenesis in citrus and longan (Wu et al., 2011; Lin and Lai, 2013) and is well known for its role in resistance (Sunkar et al., 2006; Jagadeeswaran et al., 2009). MiR398 was proposed to be directly linked to the plant stress regulatory network and regulates plant responses to oxidative stress, water deficit, salt stress, abscisic acid stress, ultraviolet stress, copper and phosphate deficiency, high sucrose, as well as bacterial infection. *MiR398* targets *Copper/zinc Superoxide Dismutase* (*CSD*) and is down-regulated under stress conditions to permit up-regulation of its target genes (Sunkar et al., 2006; Jagadeeswaran et al., 2009). The negative correlation of the expression pattern of *cre-miR398b* and *cre-CSD* was also found in our experiment, indicating that the regulation of *CSD* by *miR398b* plays important roles in the *in vitro* conservation of ponkan (Figure 8). The change of expression levels of *cre-miR398b* and its target *cre-CSD* in *in vitro* plantlet during the conversation of *C. reticulata* manifested that stress did exist in the preservation of *in vitro* plantlet and miRNAs acted as a stress-regulator.

Overall, we reported the data on miRNAs in *in vitro* plant materials and found that both stress-associated and development-associated miRNAs show similar expression patterns during the plant *in vitro* morphogenesis and preservation. The miRNAs are considered to be involved in the development via regulation of the target genes and function in the conservation of ponkan germplasm.

## Author contributions

ZL, RG, and XC designed research; RG and XC performed research and wrote the paper, and they contributed equally to this study; ZL, RG, XC, YL, XX, and MT analyzed data. ZL, XX, YL, and MT participated in the sequence analysis and helped to modify the manuscript. All authors have read and approved the manuscript for publication.

### Conflict of interest statement

The authors declare that the research was conducted in the absence of any commercial or financial relationships that could be construed as a potential conflict of interest. The reviewer Jeng-Shange Lin and handling Editor Keqiang Wu declared their shared affiliation, and the handling Editor states that, nevertheless, the process met the standards of a fair and objective review.

## Supplementary material

Supplementary Table S1 **Known miRNA in ponkan sRNA library**.

Supplementary Table S2 **Novel miRNA in ponkan sRNA library**.

Supplementary Table S3 **Enriched analysis of gene ontology annotation of ponkan miRNA**.

Supplementary Table S4 **Primers for qPCR**.

## References

[B1] Alvarez-Garcia I. Miska E. A. (2005). MicroRNA functions in animal development and human disease. Development 132, 4653–4662. 10.1242/dev.02073 16224045

[B2] Barakat A. Wall P. K. Diloreto S. dePamphilis C. W. Carlson J. E. (2007). Conservation and divergence of microRNAs in Populus. BMC Genomics 8:481. 10.1186/1471-2164-8-481 18166134 PMC2270843

[B3] Barciszewska-Pacak M. Milanowska K. Knop K. Bielewicz D. Nuc P. Plewka P.. (2015). *Arabidopsis* microRNA expression regulation in a wide range of abiotic stress responses. Front. Plant Sci. 6:410. 10.3389/fpls.2015.00410 26089831 PMC4454879

[B4] Bartel D. P. (2004). MicroRNAs: genomics, biogenesis, mechanism, and function. Cell 116, 281–297. 10.1016/S0092-8674(04)00045-5 14744438

[B5] Bencivenga S. Simonini S. Benková E. Colombo L. (2012). The transcription factors *BEL1* and *SPL* are required for cytokinin and auxin signaling during ovule development in *Arabidopsis*. Plant Cell 24, 2886–2897. 10.1105/tpc.112.100164 22786869 PMC3426121

[B6] Chen R. Zheng H. Zhang H. (2009). Identification of microRNAs in wild soybean (*Glycine soja*). J. Integr. Plant Biol. 51, 1071–1079. 10.1111/j.1744-7909.2009.00887.x 20021554

[B7] Chen X. (2005). MicroRNA biogenesis and function in plants. FEBS Lett. 579, 5923–5931. 10.1016/j.febslet.2005.07.071 16144699 PMC5127707

[B8] Chen X. D. (2011). Germplasm Conservation and microRNA Identification of in Vitro Plantlets in Citrus Trees. Thesis, Fujian Agriculture and Forestry University. Available online at: http://d.g.wanfangdata.com.cn/Thesis_Y1878643.aspx (Accessed August 24, 2011).

[B9] Chuck G. Candela H. Hake S. (2009). Big impacts by small RNAs in plant development. Curr. Opin. Plant Biol. 12, 81–86. 10.1016/j.pbi.2008.09.008 18980858

[B10] Donaire L. Pedrola L. de la Rosa R. Llave C. (2011). High-throughput sequencing of RNA silencing-associated small RNAs in olive (*Olea europaea* L.). PLoS ONE 6:e27916. 10.1371/journal.pone.0027916 22140484 PMC3225373

[B11] Ha M. Lu J. Tian L. Ramachandrand V. Kasschaue K. D. Chapmane E. J.. (2009). Small RNAs serve as a genetic buffer against genomic shock in *Arabidopsis* interspecific hybrids and allopolyploids. Proc. Natl. Acad. Sci. U.S.A. 106, 17835–17840. (Ha et al., 2009) 10.1073/pnas.0907003106 19805056 PMC2757398

[B12] Hsieh L. C. Lin S. I. Shih A. C. C. Chen J. W. Lin W. Y. Tseng C. Y.. (2009). Uncovering small RNA-mediated responses to phosphate deficiency in *Arabidopsis* by deep sequencing. Plant physiol. 151, 2120–2132. 10.1104/pp.109.147280 19854858 PMC2785986

[B13] Hwang E. Shin S. Yu B. Byun M. O. Kwon H. B. (2011). MiR171 family members are involved in drought response in *Solanum tuberosum*. J. Plant Biol. 54, 43–48. 10.1007/s12374-010-9141-8

[B14] Jagadeeswaran G. Saini A. Sunkar R. (2009). Biotic and abiotic stress down-regulate *miR398* expression in *Arabidopsis*. Planta 229, 1009–1014. 10.1007/s00425-009-0889-3 19148671

[B15] Jones-Rhoades M. W. Bartel D. P. (2004). Computational identification of plant microRNAs and their targets, including a stress-induced miRNA. Mol. Cell 14, 787–799. 10.1016/j.molcel.2004.05.027 15200956

[B16] Jones-Rhoades M. W. Bartel D. P. Bartel B. (2006). MicroRNAs and their regulatory roles in plants. Annu. Rev. Plant Biol. 57, 19–53. 10.1146/annurev.arplant.57.032905.105218 16669754

[B17] Juarez M. T. Kui J. S. Thomas J. Heller B. A. Timmermans M. C. (2004). MicroRNA-mediated repression of rolled leaf1 specifies maize leaf polarity. Nature 428, 84–88. 10.1038/nature02363 14999285

[B18] Katiyar A. Smita S. Muthusamy S. K. Chinnusamy V. Pandey D. M. Bansal K. C. (2015). Identification of novel drought-responsive microRNAs and trans-acting siRNAs from *Sorghum bicolor* (L.) Moench by high-throughput sequencing analysis. Front. Plant Sci. 6:506. 10.3389/fpls.2015.00506 26236318 PMC4504434

[B19] Kiełbasa S. M. Blüthgen N. Fähling M. Mrowka R. (2010). Targetfinder. org: a resource for systematic discovery of transcription factor target genes. Nucleic Acids Res. 38(Suppl. 2), W233–W238. 10.1093/nar/gkq374 20460454 PMC2896086

[B20] Kinoshita N. Wanga H. Kasaharac H. Liu J. MacPherson C. Machid Y.. (2012). IAA-Ala Resistant3, an evolutionarily conserved target of miR167, mediates *Arabidopsis* root architecture changes during high osmotic stress. Plant Cell 24, 3590–3602. 10.1105/tpc.112.097006 22960911 PMC3480289

[B21] Li R. Li Y. Kristiansen K. Wang J. (2008). SOAP: short oligonucleotide alignment program. Bioinformatics 24, 713–714. 10.1093/bioinformatics/btn025 18227114

[B22] Liang C. Zhang X. Zou J. Xu D. Su F. Ye N. (2010). Identification of miRNA from *Porphyra yezoensis* by high-throughput sequencing and bioinformatics analysis. PLoS ONE 5:e10698. 10.1371/journal.pone.0010698 20502668 PMC2873431

[B23] Lin Y. L. Lai Z. X. (2013). Comparative analysis reveals dynamic changes in miRNAs and their targets and expression during somatic embryogenesis in longan (*Dimocarpus longan* Lour.) PloS ONE 8:e60337. 10.1371/journal.pone.0060337 23593197 PMC3623967

[B24] Liu Y. Wang L. Chen D. Wu X. Huang D. Chen L.. (2014). Genome-wide comparison of microRNAs and their targeted transcripts among leaf, flower and fruit of sweet orange. BMC Genomics 15:695. 10.1186/1471-2164-15-695 25142253 PMC4158063

[B25] Luan Y. Wang W. Liu P. (2014). Identification and functional analysis of novel and conserved microRNAs in tomato. Mol. Biol. Rep. 41, 5385–5394. 10.1007/s11033-014-3410-4 24844213

[B26] Meng F. Liu H. Wang K. Liu L. Wang S. Zhao Y.. (2013). Development-associated microRNAs in grains of wheat (*Triticum aestivum* L.). BMC Plant Biol. 13:140. 10.1186/1471-2229-13-140 24060047 PMC4015866

[B27] Meyers B. C. Axtell M. J. Bartel B. Bartel D. P. Baulcombe D. Bowman J. L.. (2008). Criteria for annotation of plant MicroRNAs. Plant Cell 20, 3186–3190. 10.1105/tpc.108.064311 19074682 PMC2630443

[B28] Morin R. D. Aksay G. Dolgosheina E. Ebhardt H. A. Magrini V. Mardis E. R.. (2008). Comparative analysis of the small RNA transcriptomes of *Pinus contorta* and *Oryza sativa*. Genome Res. 18, 571–584. 10.1101/gr.6897308 18323537 PMC2279245

[B29] Moxon S. Jing R. Szittya G. Schwach F. Pilcher R. L. R. Moulton V.. (2008). Deep sequencing of tomato short RNAs identifies microRNAs targeting genes involved in fruit ripening. Genome Res. 18, 1602–1609. 10.1101/gr.080127.108 18653800 PMC2556272

[B30] Nawrocki E. P. Burge S. W. Bateman A. Daub J. Eberhardt R. Y. Eddy S. R.. (2014). Rfam 12.0: updates to the RNA families database. Nucleic Acids Res. 43, D130–D137. 10.1093/nar/gku1063 25392425 PMC4383904

[B31] Ori N. Cohen A. R. Etzioni A. Brand A. Yanai O. Shleizer S.. (2007). Regulation of LANCEOLATE by miR319 is required for compound-leaf development in tomato. Nat. Genet. 39, 787–791. 10.1038/ng2036 17486095

[B32] Schwarz S. Grande A. V. Bujdoso N. Saedler H. Huijser P. (2008). The microRNA regulated SBP-box genes *SPL9* and *SPL15* control shoot maturation in *Arabidopsis*. Plant Mol. Biol. 67, 183–195. 10.1007/s11103-008-9310-z 18278578 PMC2295252

[B33] Song C. Wang C. Zhang C. Korir N. Yu H. Ma Z.. (2010). Deep sequencing discovery of novel and conserved microRNAs in trifoliate orange (*Citrus trifoliata*). BMC Genomics 11:431. 10.1186/1471-2164-11-431 20626894 PMC2996959

[B34] Sun G. (2012). MicroRNAs and their diverse functions in plants. Plant Mol. Biol. 80, 17–36. 10.1007/s11103-011-9817-6 21874378

[B35] Sunkar R. Jagadeeswaran G. (2008). *In silico* identification of conserved microRNAs in large number of diverse plant species. BMC Plant Biol. 8:37. 10.1186/1471-2229-8-37 18416839 PMC2358906

[B36] Sunkar R. Kapoor A. Zhu J. K. (2006). Posttranscriptional induction of two Cu/Zn superoxide dismutase genes in *Arabidopsis* is mediated by downregulation of miR398 and important for oxidative stress tolerance. Plant Cell 18, 2051–2065. 10.1105/tpc.106.041673 16861386 PMC1533975

[B37] Szittya G. Moxon S. Santos D. M. Jing R. Fevereiro M. Moulton V.. (2008). High-throughput sequencing of *Medicago truncatula* short RNAs identifies eight new miRNA families. BMC Genomics 9:593. 10.1186/1471-2164-9-593 19068109 PMC2621214

[B38] Tsukaya H. (2006). Mechanism of leaf shape determination. Annu. Rev. Plant Biol. 57, 477–496. 10.1146/annurev.arplant.57.032905.105320 16669771

[B39] Wang J. W. Schwab R. Czech B. Mica E. Weigel D. (2008). Dual effects of miR156-targeted *SPL* genes and *CYP78A5/KLUH* on plastochron length and organ size in *Arabidopsis thaliana*. Plant Cell 20, 1231–1243. 10.1105/tpc.108.058180 18492871 PMC2438454

[B40] Wang L. Liu H. Li D. Chen H. (2011). Identification and characterization of maize microRNAs involved in the very early stage of seed germination. BMC Genomics 12:154. 10.1186/1471-2164-12-154 21414237 PMC3066126

[B41] Wang L. Mai Y. Zhang Y. Luo Q. Yang H. Q. (2010). MicroRNA171c-targeted *SCL6-II, SCL6-III*, and *SCL6-IV* genes regulate shoot branching in *Arabidopsis*. Mol. Plant. 3, 794–806. 10.1093/mp/ssq042 20720155

[B42] Wei L. Q. Yan L. F. Wang T. (2011). Deep sequencing on genome-wide scale reveals the unique composition and expression patterns of microRNAs in developing pollen of *Oryza sativa*. Genome Biol. 12:R53. 10.1186/gb-2011-12-6-r53 21679406 PMC3218841

[B43] Wu H. J. Ma Y.-K. Chen T. Wang M. Wang X. J. (2012). PsRobot: a web-based plant small RNA meta-analysis toolbox. Nucleic Acids Res, 40, W22–W28. 10.1093/nar/gks554 22693224 PMC3394341

[B44] Wu L. Zhou H. Y. Zhang Q. Q. Zhang J. G. Ni F. R. Liu C.. (2010). DNA methylation mediated by a microRNA pathway. Mol. Cell 38, 465–475. 10.1016/j.molcel.2010.03.008 20381393

[B45] Wu M. F. Tian Q. Reed J. W. (2006). *Arabidopsis* microRNA167 controls patterns of *ARF6* and *ARF8* expression, and regulates both female and male reproduction. Development 133, 4211–4218. 10.1242/dev.02602 17021043

[B46] Wu X. M. Kou S. J. Liu Y. L. Fang Y. N. Xu Q. Guo W. W. (2015). Genomewide analysis of small RNAs in nonembryogenic and embryogenic tissues of citrus: microRNA−and siRNA−mediated transcript cleavage involved in somatic embryogenesis. Plant Biotechnol. J. 13, 383–394. 10.1111/pbi.12317 25615015

[B47] Wu X. M. Liu M. Y. Ge X. X. Xu Q. Guo W. W. (2011). Stage and tissue-specific modulation of ten conserved miRNAs and their targets during somatic embryogenesis of Valencia sweet orange. Planta 233, 495–505. 10.1007/s00425-010-1312-9 21103993

[B48] Xie S. Li X. Y. Liu T. Cao J. H. Zhong Q. Zhao S. H. (2011). Discovery of porcine microRNAs in multiple tissues by a Solexa deep sequencing approach. PLoS ONE 6:e16235. 10.1371/journal.pone.0016235 21283541 PMC3026822

[B49] Xu Q. Liu Y. Zhu A. Wu X. Ye J. Yu K.. (2010). Discovery and comparative profiling of microRNAs in a sweet orange red-flesh mutant and its wild type. BMC Genomics 11:246. 10.1186/1471-2164-11-246 20398412 PMC2864249

[B50] Yao Y. Guo G. Ni Z. Sunkar R. Du J. Zhu J. K.. (2007). Cloning and characterization of microRNAs from wheat (*Triticum aestivum* L.). Genome Biol. 8:R96. 10.1186/gb-2007-8-6-r96 17543110 PMC2394755

[B51] Yin Z. J. Shen F. F. (2010). Identification and characterization of conserved microRNAs and their target genes in wheat *(Triticum aestivum*). Genet. Mol. Res. 9, 1186–1196. 10.4238/vol9-2gmr805 20589616

[B52] Yu N. Niu Q. Ng K. Chua N. (2015). The role of miR156/SPLs modules in *Arabidopsis* lateral root development. Plant J. 83, 673–685. 10.1111/tpj.12919 26096676

[B53] Zhang J. Xu Y. Huan Q. Chong K. (2009). Deep sequencing of Brachypodium small RNAs at the global genome level identifies microRNAs involved in cold stress response. BMC Genomics 10:449. 10.1186/1471-2164-10-449 19772667 PMC2759970

[B54] Zhao C. Z. Xia H. Frazier T. P. Yao Y. Y. Bi Y. P. Li A. Q.. (2010). Deep sequencing identifies novel and conserved microRNAs in peanuts (*Arachis hypogaea* L.). BMC Plant Biol. 10:3. 10.1186/1471-2229-10-3 20047695 PMC2826338

