# Supplementary material for: Identification of Novel and Conserved miRNAs in Leaves of In vitro Grown Citrus reticulata “Lugan” Plantlets by Solexa Sequencing
Source: Front Plant Sci. 2016 Jan 8;6:1212. doi: 10.3389/fpls.2015.01212 (PMC4705231; doi:10.3389/fpls.2015.01212)
Supplement: Supplementary Table S4 — Primers for qPCR. [file Table4.DOC]

Table 4. Primers for qPCR

| MiRNA name | Primers (5'to3') |
| --- | --- |
| *cre-miR156a* | GCATGACAGAAGAGAGTGAGCAC |
| *cre-miR159b* | CTTTGGATTGAAGGGAGCTCTT |
| *cre-miR160a* | CTGGCTCCCTGTATGCCA |
| *cre-miR166a* | GACCAGGCTTCATTCCCC |
| *cre-miR167a* | GAAGCTGCCAGCATGATCTA |
| *cre-miR168a* | TTGGTGCAGGTCGGGAA |
| *cre-miR171* | ATTGAGCCGCGTCAATATCTCC |
| *cre-miR398b* | AATGTGTTCTCAGGTCACCCCTG |
| *U6 snRNA* | CGATACAGAGAAGATTAGCATGG |
| *SPL9* | ACAAGTGTCTCCAAGCAGG |
|  | CGATACTGGTTGAGTGACG |
| *MYB33* | CAAGTTTGATGAACGAGAAC |
|  | TGTTTCCACCACAAGTTG |
| *ARF10* | TGAAGCGTGTTAGCCCA |
|  | TCCCATAGTCAGCAGACAAGA |
| *ARF6* | AGAGTGCACCAAGAACTGTTC |
|  | GTTAGTGGCGGTAGTAATTG |
| *SCL6* | CGAAGCAGAAGTTGGTAAACG |
|  | TTCACAGCAGTTGCCTCAC |
| *CSD* | TCCAGTGGGAGACAAGTCAC |
|  | CAGCACCTCTATGGCTATTAGG |
